# Dual RNA Sequencing Analysis of *Bacillus amyloliquefaciens* and *Sclerotinia sclerotiorum* During Infection of Soybean Seedlings by *S. sclerotiorum* Unveils Antagonistic Interactions

**DOI:** 10.3389/fmicb.2022.924313

**Published:** 2022-06-23

**Authors:** Yunqing Cheng, Xin Gao, Hongli He, Xingzheng Zhang, Ran Wang, Jianfeng Liu

**Affiliations:** Jilin Provincial Key Laboratory of Plant Resource Science and Green Production, Jilin Normal University, Siping, China

**Keywords:** soybean, RNA sequencing, *Sclerotinia sclerotiorum*, *Bacillus amyloliquefaciens*, antagonism

## Abstract

Soybean Sclerotinia stem rot is caused by *Sclerotinia sclerotiorum* infection, which causes extensive and severe damage to soybean production. Here, we isolated and patented a *Bacillus amyloliquefaciens* strain, and used it to verify the antagonistic effect of *B. amyloliquefaciens* on *S. sclerotiorum* and to explore the possible underlying mechanism. First, we conducted a plate confrontation experiment using the two microbes. Then, inoculation of soybean (*Glycine max*) seedlings with *S. sclerotiorum* (Gm-Ss), *B. amyloliquefaciens* (Gm-Ba), and their combination (Gm-Ba-Ss) was performed, followed by dual RNA sequencing analysis. Plate confrontation and inoculation experiments showed that *B. amyloliquefaciens* significantly antagonized *S. sclerotiorum* growth. The average number of fragments per kilobase of transcript per million fragments mapped of *S. sclerotiorum* transcripts in Gm-Ss and Gm-Ba-Ss inoculation treatments were 117.82 and 50.79, respectively, indicating that *B. amyloliquefaciens* strongly inhibited gene expression of *S. sclerotiorum*. In contrast, the average number of fragments per kilobase of transcript per million fragments mapped of *B. amyloliquefaciens* transcripts in Gm-Ba and Gm-Ba-Ss inoculation treatments were 479.56 and 579.66, respectively, indicating that *S. sclerotiorum* promoted overall gene expression in *B. amyloliquefaciens*. For *S. sclerotiorum*, 507 upregulated and 4,950 downregulated genes were identified among 8,975 genes in the paired comparison Gm-Ba-Ss vs. Gm-Ss. These differentially expressed genes (DEGs) were significantly enriched in the ribosome (ko03010) KEGG pathway. Additionally, for *B. amyloliquefaciens*, 294 upregulated and 178 downregulated genes were identified among all 3,154 genes in the paired comparison Gm-Ba-Ss vs. Gm-Ba, and these DEGs were mainly and significantly enriched in metabolism-related KEGG pathways, including the citrate cycle (ko00020) and carbon metabolism (ko01200). We concluded that *B. amyloliquefaciens* inhibits the expression of genes encoding the ribosomal subunit of *S. sclerotiorum*, resulting in protein synthesis inhibition in *S. sclerotiorum*, and thus had a strong antagonistic effect on the fungus. This study provides a scientific basis for the biological control of *S. sclerotiorum* by *B. amyloliquefaciens*.

## Introduction

*Sclerotinia sclerotiorum* is an important plant pathogenic fungus that causes stem rot in vegetable crops. Its host range is very wide, infecting many monocotyledons and dicotyledons, thus it could easily cause an epidemic (Clarkson et al., 2003; Li et al., 2018). Indeed, it is extremely dangerous and difficult to control, causing extensive and heavy yield losses in soybean production in countries such as China, Brazil, Canada, the United States, and South Africa. During the stem rot epidemic, soybean yield can be reduced by as much as 20–30%, and more than 50% of severely affected plots can be completely lost (Zhao et al., 2006; Dong et al., 2008; Li and Zhang, 2014). After infection by *S. sclerotiorum*, the content of crude fat, crude protein, and even the total amount of amino acids in soybean decreases, thereby affecting soybean quality (Zhang et al., 2008). At present, the prevention and control of Sclerotinia disease around the world mainly depends on chemical pesticides. However, chemical control is expensive, it causes acute environment hazards, and the level of control achieved is never ideal (Wang et al., 2020). Therefore, research and development of biological control technologies for soybean sclerotinia is very important for soybean stem rot management.

Biological control technology for *S. sclerotiorum* has made some important progress. For example, some studies have shown that *Coniothyrium minitans* with hyperparasitic effects, can be used to reduce the incidence of Sclerotinia disease (Gao et al., 2006; Mcquilken et al., 2010; Zeng et al., 2012). In addition, *Trichoderma viride* (Sumida et al., 2018), *Trichoderma harzianum* (Chitrampalam et al., 2008; Zeng et al., 2012; Sumida et al., 2018), *Bacillus subtilis* (Zhang et al., 2006), *Streptomyces avermitilis* (Ge et al., 2017), and *Streptomyces hygroscopicus* (Ge et al., 2017) have antagonistic effects on *S. sclerotiorum*. *Sclerotinia sclerotiorum* hypovirulence-associated DNA virus 1 (SsHADV-1) is a single-stranded DNA virus and can convert its host, *S. sclerotiorum*, from a typical necrotrophic pathogen to a beneficial endophytic fungus and reduce the degree of disease (Kraberger et al., 2013; Zhang et al., 2020). The above research not only provides a theoretical basis for the biological control of soybean Sclerotinia but lays a foundation for the development of biocontrol agents in the future as well. However, these bacterial and viral biocontrol agents have not been utilized for field control of *S. sclerotiorum*; their effectiveness and production cost may be a major reason for their limited application in the field. To date, the application of chemical pesticides remains the major method for *S. sclerotiorum* control. Therefore, screening new, cost-effective Sclerotinia antagonistic microorganisms, and exploring the interaction mechanism of soybean sclerotinia antagonists have important theoretical and practical value for the development of biological control products for Sclerotinia.

Here, we isolated and patented a *Bacillus amyloliquefaciens* strain with universal resistance to fungi. This strain effectively inhibited the growth of *S. sclerotiorum*. Inoculation of *S. sclerotiorum* alone on the stems of soybean seedlings resulted in wilting and death. In contrast, inoculation with *B. amyloliquefaciens* alone or in combination with *S. sclerotiorum* had no adverse effects on soybean seedlings and either stopped or killed the fungus in co-culture. Based on these results, *B. amyloliquefaciens* may have potential as a biological control product against *S. sclerotiorum*. To elucidate the mechanism of interaction between *S. sclerotiorum* and *B. amyloliquefaciens*, we used dual RNA-seq technology to analyze gene expression changes in *B. amyloliquefaciens* and *S. sclerotiorum* after inoculation of soybean seedling stems with either *S. sclerotiorum* alone, or mixed with *B. amyloliquefaciens*. Our data provided new insights for the development of practical biological control products of soybean *S. sclerotiorum*.

## Materials and Methods

### Verification of Antagonistic Effect of *Bacillus amyloliquefaciens* Against *Sclerotinia sclerotiorum* in Plate Experiments

The experimental microorganisms included *S. sclerotiorum* (China General Microbiological Culture Collection Center, CGMCC, NO 3.7083) and *B. amyloliquefaciens* (CGMCC, NO 20507). Preserved *S. sclerotiorum* and *B. amyloliquefaciens* were inoculated and activated in the potato dextrose agar (PDA) medium before the antagonism experiment. The antagonistic effect of *B. amyloliquefaciens* against *S. sclerotiorum* was measured on PDA plates. Five millimeter plugs of *S. sclerotiorum*, taken from an actively growing culture, and then the *B. amyloliquefaciens* strain culture (1 × 10^9^ CFU/ml, 20 μl) was inoculated at four corners of the plate, equidistant from the center. The cultures were maintained in a 25°C incubator to observe the growth and antagonism between *S. sclerotiorum* and *B. amyloliquefaciens*. The antagonistic experiment included three biological replicates, each containing three plates.

### Antagonistic Effects of *Bacillus amyloliquefaciens* Against *Sclerotinia sclerotiorum* in Stems of Soybean Seedlings

Soybean (Cultivar Williams 82, w82) seeds were sown in plastic pots containing pure humus and cultured in an artificial climate chamber at temperature, 28°C, under a photosynthetic photon flux density of 60 μmol m^−2^ s^−1^ and a 16 h light/8 h dark light regime. After 25 d of sowing, 40 pots with seedlings at the soybean V4 stage (Fehr and Caviness, 1977) were selected for the inoculation experiment. The microbial inoculation position was in the middle of the two lowest nodes of the seedling stems. The experiments included four treatments: (1) soybean seedling wounded with a scalpel and not inoculated was used as the control; (2) *S. sclerotiorum,* Ss; (3) *B. amolyliquefacie,* Ba; (4) *S. sclerotiorum* mixed with *B. amyloliquefaciens*, Ss + Ba. The inoculation treatments were performed as follows. Five millimeter plugs of *S. sclerotiorum*, taken from an actively growing culture, were inoculated into 6 cm diameter Petri dishes containing PDA medium. After 1 week of culture, 20 plugs without medium (0.2 cm × 0.2 cm) were collected from the inner walls of the Petri dishes using a scalpel, and inoculated into a 300-mL potato dextrose broth (PDB), followed by culture for 48 h at 28°C with constant shaking (200 rpm), generating approximately 20 mycelial masses with diameters of approximately 0.6 cm. One mycelial mass was used for subsequent Ss inoculation of one soybean seedling. *B. amyloliquefaciens* culture, with OD_600_ of 0.1, was inoculated in a 200-mL PDB medium and cultured for 48 h at 28°C with constant shaking (200 rpm) till OD_600_ value reached 4. Then, 5.0 ml *B. amyloliquefaciens* culture was used for subsequent Ba inoculation materials on one soybean seedling. The mixtures of 5.0 ml *B. amyloliquefaciens* culture and one mycelial mass of *S. sclerotiorum* was used for subsequent Ss + Ba inoculation materials on one soybean seedling. Before inoculation on each soybean seedling, all inoculation materials were centrifuged at 5000 × *g* for 5 min, and the precipitates were centrifugally washed with sterile saline at 5000 × *g* for 5 min. A longitudinal wound, approximately 2 cm in length, was made on the stem of soybean seedlings using a scalpel, and the wound depth was less than 20% of the stem diameter. The three treatments (Ss, Ba, and Ss + Ba) were administered by inoculating with *S. sclerotiorum* mycelial mass, *B. amyloliquefaciens* sediment mentioned above, and their mixture, respectively. After inoculation, the inoculated part of the stem was wrapped in a plastic fresh-keeping film. The incidence of Sclerotinia disease after the treatment was observed daily. These experiments were repeated to facilitate RNA extraction. On the second day after inoculation, the stem segments of soybean seedlings with the above three treatments (Ss, Ba, and Ss + Ba) were used as experimental materials. A 2 cm segment of the stem, surrounding the inoculation site, was excised for RNA extraction. Each treatment included three biological replicates, and each replicate included three stem segments of soybean seedlings.

### Inhibition of *Sclerotinia sclerotiorum* by Extracellular Products and Broken *Bacillus amyloliquefaciens* Bacteria

To elucidate the inhibitory mechanism of *B. amyloliquefaciens* against *S. sclerotiorum*, extracellular products and burst bacterial cells were prepared using the following protocol. *B. amyloliquefaciens* was inoculated into 10 ml PDB and cultured at 28°C with 200 rpm constant shaking for 24 h, followed by centrifugation at 5200 × g for 10 min at 4°C. The supernatant was filtered through a 0.22 μm filtration membrane to acquire the extracellular products. Sterile normal saline (5 ml) was added to the precipitate. The samples were frozen −80°C for 20 min and placed in a water bath at 37°C for 20 min. Freezing and thawing were repeated four times, and the solution with burst bacterial cells was centrifuged at 5200 × *g* for 10 min at 4°C. The supernatant was filtered through a 0.22 μm filtration membrane to acquire the burst bacterial cells. *S. sclerotiorum* was cultured on fresh PDA plates at 28°C in the dark for a week. A 6 mm agar plug of *S. sclerotiorum* from an actively growing PDA culture was transferred to the center of a fresh PDA plate. The sterile normal saline, extracellular products, and burst *B. amyloliquefaciens* cells (20 μl) were inoculated at two corners of the plate, equidistant from the center. Finally, the plates were incubated at 28°C and routinely monitored for mycelial spread. Each treatment included three biological replicates, and each replicate included five plates.

### Library Preparation and Sequencing

To investigate the antagonistic interactions of *B. amyloliquefaciens* during *Sclerotinia sclerotiorum* infection of soybean (*Glycine max*) seedlings, we constructed nine digital gene expression (DGE) profiling libraries, namely Gm-Ss-1, Gm-Ss-2, Gm-Ss-3, Gm-Ba-1, Gm-Ba-2, Gm-Ba-3, Gm-Ba-Ss-1, Gm-Ba-Ss-2, and Gm-Ba-Ss-3. Total RNA was extracted from the stem segments of soybeans using the RNA EasySpin Isolation System (Aidlab Biotech, Beijing, China). RNA degradation and contamination were monitored using 1% agarose gel electrophoresis. RNA purity was assessed using a NanoPhotometer spectrophotometer (IMPLEN, CA, United States). RNA integrity was assessed using the RNA Nano 6,000 Assay Kit on the Agilent Bioanalyzer 2,100 system (Agilent Technologies, CA, United States). A total amount of 2 μg RNA per sample was used as the input material for RNA sample preparation. Sequencing libraries were generated using the NEBNext^®^Ultra^™^ RNA Library Prep Kit for Illumina^®^(#E7530L, NEB, United States) following manufacturer recommendations, and index codes were added to attribute sequences to each sample. Briefly, rRNA was removed from total RNA using the Ribo-Zero rRNA Removal Kit to purify mRNA. Fragmentation was conducted using divalent cations under elevated temperatures in NEBNext First Strand Synthesis Reaction Buffer (5X). First-strand cDNA was synthesized using random hexamer primers and RNase H. Second-strand cDNA synthesis was subsequently performed using buffer, dNTPs, DNA polymerase I, and RNase H. The library fragments were purified using AMPure XP beads and eluted with EB buffer, followed by terminal repair, A-tailing, and adapter addition. The products were retrieved by agarose gel electrophoresis. The UNG enzyme was used to digest the second strand of cDNA, and PCR was performed, after which the library was completed. Then, Qubit4.0 was used for preliminary quantification, and the library was diluted to 1.0 ng/μL. Library quality was assessed using an Agilent Bioanalyzer 2100system. Library preparations were sequenced on an Illumina Novaseq6000 platform, and paired-end reads were generated using the PE150 (double-terminal 150 bp) sequencing protocol.

### Bioinformatics Analysis of Transcriptome Data

To ensure the accuracy of subsequent bioinformatics analysis, raw data were filtered using the cutadapt v2.7.^1^ To obtain high quality clean reads, adapter sequences, reads with quality value less than Q20, reads with more than 10% N, and reads of less than 25 bp after trimming were removed. The index of the reference genome was constructed using HISAT2 (v 2.1.0), and the clean reads were aligned to the reference sequence using Hisat2 (v 2.1.0) to calculate the gene alignment rate. To obtain the sequence information of one particular species, the sequences of other species need to be filtered to eliminate the background interference. For example, in case of the Gm-Ba-Ss treatment, the extracted RNA was from three species, including *G. max*, *S. sclerotiorum*, and *B. amyloliquefaciens*. If we want to obtain the clean reads of *B. amyloliquefaciens*, sequences of *G. max and S. sclerotiorum* must be filtered out using the following strategy. The data were mapped to the reference genome of *G. max*, and the matched sequence data were discarded; the remaining data were used for subsequent analysis. Then, similarly, the data were mapped to the reference genome of *S. sclerotiorum*, and the matched sequence data were discarded; the remaining data were used for subsequent analysis. Finally, the data were mapped to the reference genome of *B. amyloliquefaciens*, and the matched data will be used as clean reads of *B. amyloliquefaciens* in Gm-Ba-Ss treatment for subsequent bioinformatics analysis. The reference genomes of *G. max*, *S. sclerotiorum*, and *B. amyloliquefaciens* can be found at https://www.soybase.org/GlycineBlastPages/blast_descriptions.php#Wm82.a4.v1.genome.nt, https://ftp.ncbi.nlm.nih.gov/genomes/all/GCF/000/146/945/GCF_000146945.2_ASM14694v2/, and https://ftp.ncbi.nlm.nih.gov/genomes/all/GCF/000/242/855/GCF_000242855.2_ASM24285v2, respectively. After sequence filtering, ‘HISAT2’^2^ was used to map the RNA-seq reads (Kim et al., 2015) and Stringtie (v1.3.3b)^3^ was used to assemble the mapped reads (Pertea et al., 2015). TransDecoder (r201311110; parameters: default) software was used to predict the coding region sequence and the corresponding amino acid sequence of the transcript. Then, HMMER (v3.3.2; parameter e-value = 0.01) was used to align the transcript sequence to Pfam (protein family) database, and the information about protein domain classification was obtained. Then, the transcript sequences were aligned to non-redundant (NR; a protein sequence database of NCBI), string (a database of predicted functional associations between proteins), Swissprot (a manually annotated and reviewed protein sequence database), and Kyoto Encyclopedia of Genes and Genomes (KEGG) functional database protein sequences using the software diamond (v2.0.8) to obtain the corresponding annotation information. The gene ontology (GO) annotation was parsed from the results of Swissprot. FPKM (fragments per kilobase of transcript per million fragments mapped) to measure the expression level of transcripts by StringTie using the maximum flow algorithm. Differentially expressed genes (DEGs) were identified using the bioconductor R package ‘DESeq2’ (Love et al., 2014). The criteria for DEGs were set as |log_2_FoldChange > 1| and a false discovery rate (FDR) < 0.05. FoldChange refers to the ratio of gene expression in the two samples, and FDR refers to the adjusted value of *p*, which was used to measure the significance of the differences. Gene set enrichment analysis (Tan and Lenhard, 2016) was performed on all genes based on their expression levels, and the gene sets of KEGG pathways and GO terms for BP (biological process), CC (cellular component), and MF (molecular function) were targeted as gene sets of interest. Genes from each group were used as background gene sets, and enriched gene sets were identified at *p* < 0.001 and FDR < 0.05.

### Quantitative qRT-PCR Analysis

The RNA EasySpin Isolation System (Aidlab Biotech, Beijing, China) was used to extract total RNA from the soybean plants using the same samples that were used for RNA-seq analysis following manufacturer instructions. In all, seven *S. sclerotiorum* DEGs in the paired comparison of Gm-Ba-Ss vs. Gm-Ss (mock treatment) and seven *B. amyloliquefaciens* DEGs in the paired comparison of Gm-Ba-Ss vs. Gm-Ba (mock treatment) were selected for qRT-PCR analysis. Primer design and qRT-PCR analysis were performed as previously described (Liu et al., 2021). Detailed primer sequences are listed in Supplementary Table S1. The *ubiquitin* and *gyrA* genes were used as reference genes in the qRT-PCR assays for *S. sclerotiorum and B. amyloliquefaciens*, respectively, and the 2^−ΔΔCt^ method was used to calculate the fold changes of the selected genes (Schmittgen and Livak, 2008).

## Results

### Antagonistic Effect of *Bacillus amyloliquefaciens* on *Sclerotinia sclerotiorum*

**Figure 1:**
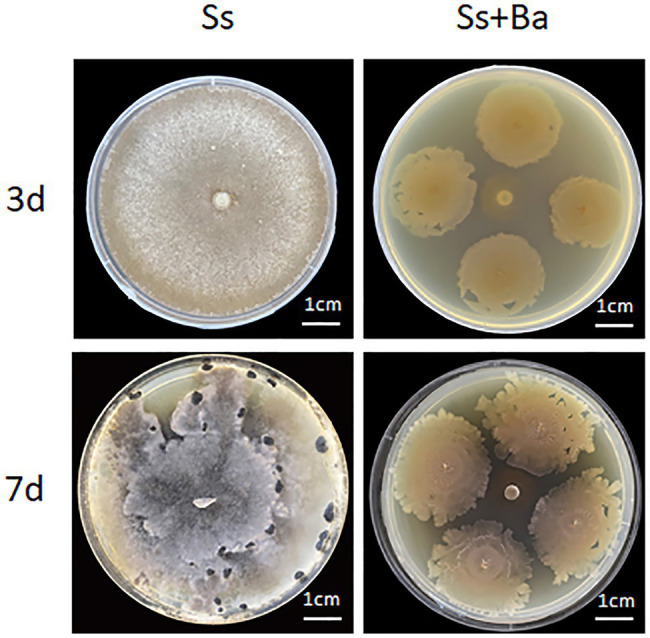
The PDA plate confrontation experiment of *Bacillus amyloliquefaciens* antagonizing the growth of *Sclerotinia sclerotiorum*. Ba, *B. amyloliquefaciens*; Ss, *S. sclerotiorum*.

To confirm the antagonistic effect of *B. amyloliquefaciens* on *S. sclerotiorum*, a PDA plate-confrontation experiment was performed. The results showed that when *S. sclerotiorum* was inoculated alone, white mycelia covered the whole plate at 3 day post-inoculation, and sclerotia were observed at 7 day post-inoculation, indicating its fast growth rate (Figure 1). In contrast, when *S. sclerotiorum* was inoculated in the center of the plate and *B. amyloliquefaciens* was inoculated around *S. sclerotiorum*, the results showed that the colony diameter of *B. amyloliquefaciens* cultured for 7d was significantly larger than that of *B. amyloliquefaciens* cultured for 3d, suggesting *B. amyloliquefaciens* also grew rapidly. Furthermore, the colony diameter of *S. sclerotiorum* on the 7th day was approximately the same as that on the third day, suggesting that *S. sclerotiorum* growth was inhibited (Figure 1). Based on the above observations, we concluded that *B. amyloliquefaciens* significantly inhibited the growth of *S. sclerotiorum* when the two microbes were inoculated simultaneously on PDA plates.

**Figure 2:**
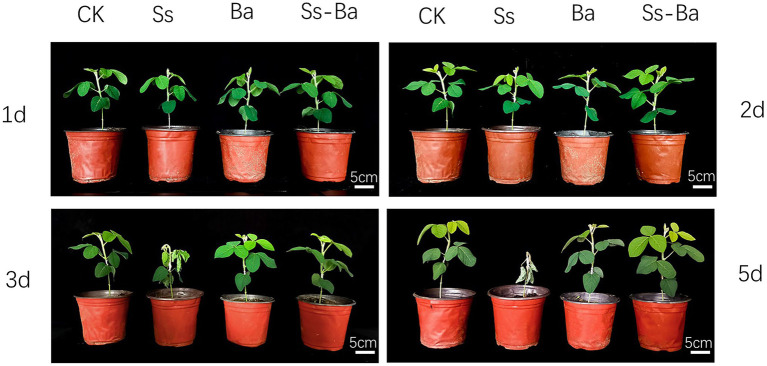
Soybean seedling inoculation experiment of *Bacillus amyloliquefaciens* antagonizing the growth of *S. sclerotiorum*. Ba, Ss, and Ba+Ss indicated that soybean seedling was inoculated with *B. amyloliquefaciens*, *S. sclerotiorum* and their mixture, respectively. Ck, soybean seedling wounded using a scalpel and not inoculated was used as the control.

To further verify the antagonistic effect of *B. amyloliquefaciens* on *S. sclerotiorum* in soybean seedlings, stem inoculation experiments were performed. The results showed that the top leaves of soybean seedlings wilted on the 3rd day post-inoculation in the Ss treatment, and all leaves appeared dehydrated and wilted on the 5th day post-inoculation (Figure 2), indicating that *S. sclerotiorum* was very aggressive on the soybean seedlings. These results were consistent with previous reports (Dong et al., 2008; Li and Zhang, 2014). Compared with controls, seedlings in the Ba treatment were not significantly different. No appreciable changes were observed on the first, second, third, or 5th day post-inoculation (Figure 2), indicating that *B. amyloliquefaciens* inoculation was not pathogenic to soybean. Similarly, in the Gm-Ba-Ss treatment, soybean seedlings showed no appreciable changes at 1, 2, 3, or 5 days post-inoculation (Figure 2). These results indicate that co-inoculation of *B. amyloliquefaciens* in soybean seedlings clearly and effectively antagonized *S. sclerotiorum*, and reduced the damage caused by *S. sclerotiorum* to soybean seedlings.

### Antagonistic Effect of *Bacillus amyloliquefaciens* Extracellular and Intracellular Products on *Sclerotinia sclerotiorum*

**Figure 3:**
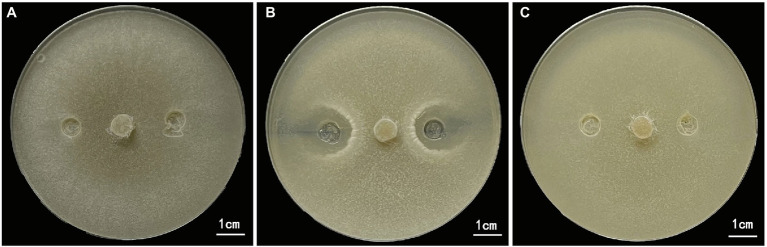
Inhibition effect of extracellular and intracellular products of *Bacillus amyloliquefaciens* on the growth of *S. sclerotida.* **(A)** Control, sterile normal saline treatment. **(B)** *B. amyloliquefaciens* extracellular products treatment. **(C)** *B. amyloliquefaciens* intracellular products treatment. Sterile normal saline, extracellular and intracellular products were inoculated at 1/4 and 3/4 of the diameter of the Petri dish.

After 10 days of culture on PDA plates, no bacteriostatic circles were formed in the margins between the *S. sclerotiorum* plug and the sterile normal saline or the burst bacteria intracellular products inoculation sites (Figures 3A,C). Conversely, in the treatment with inoculated extracellular products of *B. amyloliquefaciens*, two symmetrical and obvious bacteriostatic circles were formed in the margins between the extracellular products and the *S. sclerotiorum* plug (Figure 3B). These results showed that extracellular products had a strong inhibitory effect on the growth of *S. sclerotiorum*, but the inhibitory effect of intracellular products of *B. amyloliquefaciens* on the growth of *S. sclerotiorum* was not obvious.

### Dual RNA Sequencing, Quality Control, and Sequence Mapping

Nine biological samples from the three treatment groups were subjected to dual RNA sequencing. A total of 1,045,658,696 raw reads were acquired, covering a length of 156,848,804,400 bp. After filtering low-quality reads, 1,045,046,606 clean reads with an average Q30 of 93.67% were generated. Detailed information on the sequencing data for each sample is provided in Supplementary Table S2. The clean data were mapped to the reference genomes of *S. sclerotiorum* and *B. amyloliquefaciens*. In all, 204,809,779 reads were mapped to the reference genome of *S. sclerotiorum*. Among these, 176,015,346 reads were mapped to the reference genome, and 28,794,433 reads were mapped to multiple locations on the genome (Supplementary Table S3). Meanwhile, a total of 9,050,195 reads were mapped to the reference genome of *B. amyloliquefaciens*. Among these, 4,131,782 reads were uniquely mapped to the reference genome, and 4,918,413 reads were mapped to multiple locations on the genome (Supplementary Table S4).

### Differences in Global Gene Expression Level Between *S. sclerotiorum* and *Bacillus amyloliquefaciens*

**Figure 4:**
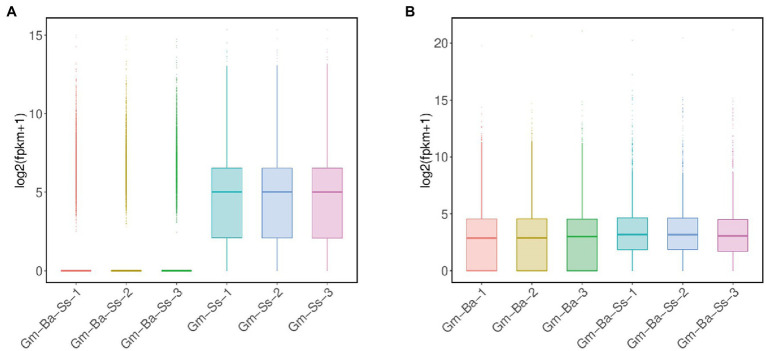
Box plot of transcript expression. **(A)** Box plot of *S. sclerotiorum* transcript expression in treatments of Ss and Ba-Ss. **(B)** Box plot of *B. amyloliquefaciens* transcript expression in treatments of Ba and Ba-Ss. Gm-Ba, Gm-Ss, Gm-Ba-Ss indicated that the *Glycine max* seedling were inoculated with *B. amyloliquefaciens*, *S. sclerotiorum* and their mixture, respectively.

A total of 14,714 transcripts of *S. sclerotiorum* transcripts were detected in the Gm-Ss and Gm-Ba-Ss treatments (Supplementary Table S5). We drew a box diagram of *S. sclerotiorum* gene expression in six biological samples from these two treatments (treatment Gm-Ss and Gm-Ba-Ss). The global gene expression level of *S. sclerotiorum* in the three biological samples of the Gm-Ss treatment was much higher than that of the Gm-Ss + Ba treatment (Figure 4A). The average FPKM of *S. sclerotiorum* transcripts in treatments Gm-Ss and Gm-Ba-Ss were calculated at 117.82 and 50.79, respectively (Supplementary Table S5). The former was much higher than the latter, which was consistent with the results shown in Figure 4A. Further, these results showed that *B. amyloliquefaciens* strongly inhibited the gene expression of *S. sclerotiorum*.

A total of 3,927 transcripts of *B. amyloliquefaciens* were detected in the Gm-Ba and Gm-Ba-Ss treatments (Supplementary Table S6). We drew a box diagram of *B. amyloliquefaciens* gene expression in six biological samples from two treatments (Ba and Ss + Ba). The global gene expression level of *B. amyloliquefaciens* in the three biological samples of the Ba treatment was lower than that in the Gm-Ba-Ss treatment (Figure 4B). The average FPKM of *B. amyloliquefaciens* transcripts in treatment Ba and Ss + Ba were calculated at 479.56 and 579.66, respectively (Supplementary Table S6). The former was lower than the latter consistently, with the results shown in Figure 4B. Further, these results suggest that *S. sclerotiorum* enhanced the overall gene expression level of *B. amyloliquefaciens* in soybean seedlings.

### DEGs in the Paired Comparisons Gm-Ba-Ss vs. Gm-Ss and Gm-Ba-Ss vs. Gm-Ba

**Figure 5:**
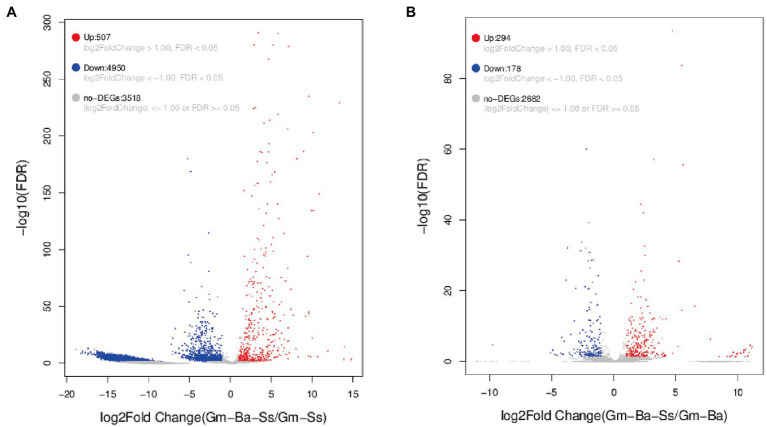
Volcano map of differentially expressed genes (DEGs). **(A)** *S. sclerotiorum* DEGs in paired comparison of Gm-Ba-Ss vs. Gm-Ss; **(B)** *B. amyloliquefaciens* in paired comparison of DEGs Gm-Ba-Ss vs. Gm-Ba. Gm-Ba, Gm-Ss, Gm-Ba-Ss indicated that the *Glycine max* seedling were inoculated with *B. amyloliquefaciens*, *S. sclerotiorum* and their mixture, respectively. Each dot represents a specific gene. Red dots indicating significantly up-regulated genes and blue dots indicating significantly down-regulated genes. Black dots are not DEGs.

A |log_2_FoldChange > 1| and a false discovery rate (FDR) < 0.05 were used to identify DEGs in the two paired comparisons (Figure 5). A volcano map of differentially expressed genes was constructed to determine the global distribution of DEGs. Thus, for *S. sclerotiorum*, 507 upregulated and 4,950 downregulated genes were identified among 8,975 genes in the paired comparison of Gm-Ba-Ss vs. Gm-Ss (Figure 5A; Supplementary Table S7). In this case, DEGs accounted for 60.80% of all genes, and downregulated DEGs accounted for 90.71% of all DEGs. These results suggest that *B. amyloliquefaciens* strongly inhibited gene expression of *S. sclerotiorum*, consistently with the results shown in Figure 4A. In turn, 294 upregulated and 178 downregulated genes were identified in *B. amyloliquefaciens* among the 3,154 genes in the paired comparison of Gm-Ba-Ss vs. Gm-Ba (Figure 5B; Supplementary Table S8). In this case, DEGs accounted for 14.97% of all identified genes, and upregulated DEGs accounted for 62.29% of all DEGs. These results confirmed that gene expression of *B. amyloliquefaciens* was promoted by *S. sclerotiorum*, consistently with the results shown in Figure 4B. Altogether, the above results confirmed that under the co-culture conditions used here for *B. amyloliquefaciens* and *S. sclerotiorum*, *B. amyloliquefaciens* strongly inhibited the gene expression of *S. sclerotiorum*, while, conversely, *S. sclerotiorum* induced the gene expression of *B. amyloliquefaciens*.

### GO Enrichment Analysis of DEGs in the Paired Comparisons Gm-Ba-Ss vs. Gm-Ss and Gm-Ba-Ss vs. Gm-Ba

**Figure 6:**
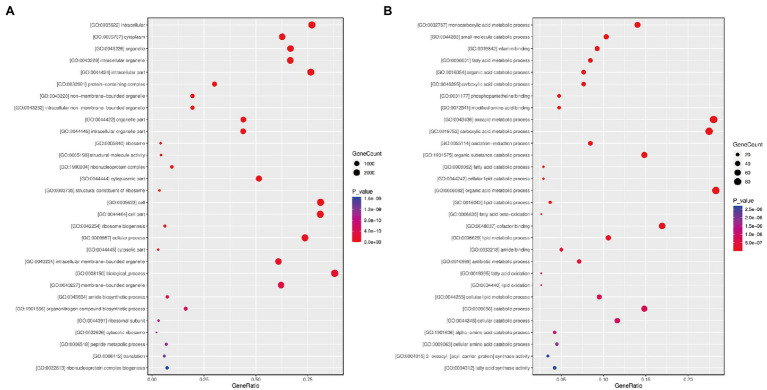
Bubble diagram of differentially expressed genes (DEGs) GO functional enrichment analysis. **(A)** *S. sclerotiorum* DEGs in paired comparison of Gm-Ba-Ss vs. Gm-Ss; **(B)** *B. amyloliquefaciens* DEGs in paired comparison of Gm-Ba-Ss vs. Gm-Ba. Gm-Ba, Gm-Ss, Gm-Ba-Ss indicated that the *Glycine max* seedling were inoculated with *B. amyloliquefaciens*, *S. sclerotiorum* and their mixture, respectively. The abscissa represents GeneRatio, that is, the proportion of the DEGs belonging to a certain GO term in all the DEGs. The larger the bubble is, the more differentially expressed genes are. Color represents the significance of enrichment, that is, *p*-value. The darker the color, the more significant the enrichment of the GO term. The color gradient on the right represents the value of *p*.

We performed GO enrichment analysis to explore the overall functions of *S. sclerotiorum* DEGs by paired comparison between the Gm-Ba-Ss and Gm-Ss treatments. All 5,457 DEGs were assigned to three categories, namely, biological processes, cellular components, and molecular functions (Figure 6A; Supplementary Table S9). In the biological process category, the most significantly enriched GO terms were GO:0042254 (ribosome biogenesis), followed by GO:0009987 (cellular process), GO:0008150 (biological process), and GO:0043604 (amide biosynthetic process; Supplementary Table S5). In turn, the most significantly enriched GO term in the cellular component category was GO:0005622 (intracellular), followed by GO:0005737 (cytoplasm), GO:0043226 (organelle), and GO:0043229 (intracellular organelle). Lastly, in the molecular function category, the most significantly enriched GO terms were GO:0005198 (structural molecule activity), GO:0003735 (structural constituent of ribosome), GO:0003723 (RNA binding), and GO:0051020 (GTPase binding).

In addition, we performed GO enrichment analysis of 472 *B. amyloliquefaciens* DEGs in the paired comparison of Gm-Ba-Ss vs. Gm-Ba (Figure 6B; Supplementary Table S10). Based on the significantly enriched GO terms, these DEGs were involved in monocarboxylic acid metabolic process (GO:0032787), small molecule catabolic process (GO:0044282), fatty acid metabolic process (GO:0006631), organic acid catabolic process (GO:0016054), and carboxylic acid catabolic process (GO:0046395). The encoded proteins were located in the spore wall (GO:0031160), cell wall (GO:0005618), external encapsulating structure (GO:0030312), proton-transporting two-sector ATPase complex (GO:0033178), and the proton-transporting ATP synthase complex (GO:0045261). Most of these DEGs had vitamin binding activity (GO:0019842), phosphopantetheine binding activity (GO:0031177), modified amino acid binding activity (GO:0072341), cofactor binding activity (GO:0048037), and amide binding activity (GO:0033218).

### KEGG Enrichment Analysis of DEGs in the Paired Comparisons Gm-Ba-Ss vs. Gm-Ss and Gm-Ba-Ss vs. Gm-Ba

KEGG pathway functional enrichment analysis was performed for all 5,457 DEGs in the paired comparison Gm-Ba-Ss vs. Gm-Ss. Although a very large number of *S. sclerotiorum* DEGs were involved in KEGG significant enrichment analysis, only one KEGG pathway, ribosome (ko03010), was significantly enriched with an adjusted *p* value of 0.0004 (Table 1). Consistent with these results, GO:0042254 (ribosome biogenesis) and GO:0003735 (structural constituent of ribosome) were identified as the most significantly enriched GO terms (Supplementary Table S5). These results suggest that *B. amyloliquefaciens* antagonism against *S. sclerotiorum* may be related to the strong inhibition of ribosome biogenesis in *S. sclerotiorum*.

**Table 1:** KEGG enrichment analysis of *S. sclerotiorum* in paired comparison of Gm-Ba-Ss vs. Gm-Ss.

TermID	Description	Category	GeneRatio	BgRatio	Enrich factor	P adjust
ko03010	Ribosome	Genetic information processing	95/1432	99/1805	1.21	0.0004
ko01200	Carbon metabolism	Metabolism	71/1432	77/1805	1.16	0.1796
ko00190	Oxidative phosphorylation	Metabolism	76/1432	83/1805	1.15	0.1796
ko01120	Microbial metabolism in diverse environments	Metabolism	124/1432	141/1805	1.11	0.2485

As for the KEGG pathway functional enrichment analysis of all 472 *B. amyloliquefaciens* DEGs in the paired comparison Gm-Ba-Ss vs. Gm-Ba, the data revealed 18 significantly enriched KEGG pathways, 17 of which were related to metabolism (Table 2). The most significantly enriched KEGG pathway was citrate cycle (ko00020), followed by carbon metabolism (ko01200), microbial metabolism in diverse environments (ko01120), butanoate metabolism (ko00650), and fatty acid degradation (ko00071). In the paired comparisons of Gm-Ba-Ss and Gm-Ba, *B. amyloliquefaciens* DEGs accounted for 14.97% of all identified genes. Collectively, these results suggest that the effect of *S. sclerotiorum* on *B. amyloliquefaciens* was mainly on metabolism.

**Table 2:** KEGG enrichment analysis of *B. amyloliquefaciens* in paired comparison of Gm-Ba-Ss vs. Gm-Ba.

TermID	Description	Category	GeneRatio	BgRatio	Enrich factor	P adjust
ko00020	Citrate cycle (TCA cycle)	Metabolism	11/169	18/1027	3.71	0.0014
ko01200	Carbon metabolism	Metabolism	26/169	74/1027	2.14	0.0014
ko01120	Microbial metabolism in diverse environments	Metabolism	41/169	142/1027	1.75	0.0014
ko00650	Butanoate metabolism	Metabolism	11/169	20/1027	3.34	0.0014
ko00071	Fatty acid degradation	Metabolism	7/169	9/1027	4.73	0.0014
ko00310	Lysine degradation	Metabolism	7/169	9/1027	4.73	0.0014
ko00620	Pyruvate metabolism	Metabolism	12/169	25/1027	2.92	0.0031
ko00190	Oxidative phosphorylation	Metabolism	13/169	29/1027	2.72	0.0035
ko00072	Synthesis and degradation of ketone bodies	Metabolism	5/169	6/1027	5.06	0.0073
ko00640	Propanoate metabolism	Metabolism	11/169	25/1027	2.67	0.0104
ko01212	Fatty acid metabolism	Metabolism	8/169	16/1027	3.04	0.0167
ko00720	Carbon fixation pathways in prokaryotes	Metabolism	10/169	23/1027	2.64	0.0167
ko00500	Starch and sucrose metabolism	Metabolism	10/169	24/1027	2.53	0.0215
ko00010	Glycolysis / Gluconeogenesis	Metabolism	11/169	28/1027	2.39	0.0215
ko00280	Valine, leucine and isoleucine degradation	Metabolism	8/169	17/1027	2.86	0.0215
ko00362	Benzoate degradation	Metabolism	5/169	8/1027	3.80	0.0271
ko00660	C5-Branched dibasic acid metabolism	Metabolism	5/169	8/1027	3.80	0.0271
ko04146	Peroxisome	Cellular Processes	4/169	6/1027	4.05	0.0495

### DEGs Verification Using qRT-PCR

**Figure 7:**
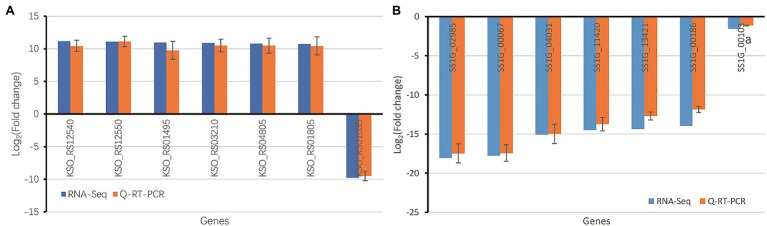
Validation of RNA-seq data using qRT-PCR **(A)** Seven randomly chosen *B. amyloliquefaciens* DEGs in paired comparison of Gm-Ba-Ss vs. Gm-Ba. **(B)** Seven randomly chosen *S. sclerotiorum* DEGs in paired comparison of Gm-Ba-Ss vs. Gm-Ss. Gm-Ba, Gm-Ss, Gm-Ba-Ss indicated that the *Glycine max* seedling were inoculated with *B. amyloliquefaciens*, *S. sclerotiorum* and their mixture, respectively.

Seven *S. sclerotiorum* DEGs in the paired comparison Gm-Ba-Ss vs. Gm-Ss, and seven *B. amyloliquefaciens* DEGs in the paired comparison Gm-Ba-Ss vs. Gm-Ss were chosen for qRT-PCR analysis. The relative changes in log_2_FoldChange measured by qRT-PCR are shown in Figure 7. As can be seen, the trends of gene expression change were consistent with the results obtained by RNA-Seq, suggesting that the results of dual RNA sequencing analysis were generally reliable.

## Discussion

Soybean Sclerotinia stem rot is a disease caused by *S. sclerotiorum* infection that can greatly reduce soybean crop yields. To date, chemical control remains the most important means of controlling infection by *S. sclerotiorum*. However, it is highly polluting of the environment and its control efficacy varies based on multiple factors including the timing of application. Alternatively, in recent years, important progress has been made in biological technology to control Sclerotinia stem rot. For example, the following microorganisms have been reported to exert antagonistic effects on *S. sclerotiorum*: *Coniothyrium minitans* (Mcquilken et al., 2010), *Trichoderma viride* (Rezende et al., 2020), *Trichoderma harzianum* (Rezende et al., 2020), *Bacillus subtilis* (Zhang et al., 2006), *Streptomyces avermitilis* (Ge et al., 2017), *Streptomyces hygroscopicus* (Ge et al., 2017), and sshadv-1 virus (Qu et al., 2021). However, the control effects of these microbes on Sclerotinia stem rot have not yet been verified in field experiments. To date, there are no reports of *B. amyloliquefaciens* antagonizing *S. sclerotiorum*, although *B. amyloliquefaciens* CH-2 has been reported to inhibit mycelial growth and sclerotia formation in *S. sclerotiorum* isolated from *Brassica campestris* (Chen et al., 2005). Our results suggest that *B. amyloliquefaciens* effectively antagonized *S. sclerotiorum* in plate confrontation experiments, and greatly alleviated the harm of *S. sclerotiorum* to soybean seedlings in the inoculation experiment reported herein. Thus, our study provides a new candidate strain for biological control of *S. sclerotiorum*.

Many studies have suggested that bacteria could produce substances that inhibit growth of other microorganisms (Arrebola et al., 2010). For example, *B. amyloliquefaciens* PPCB004 was characterized for its antifungal activity against seven selected fungal postharvest pathogens of citrus, including *Alternaria citri*, *Botryosphaeria* sp., and *Colletotrichum gloeosporioides*. The iturin family of lipopeptides found in PPCB004 is reportedly responsible for the antagonism of *B. amyloliquefaciens* against these fungi (Arrebola et al., 2010). Similarly, *B. amyloliquefaciens* L1 has a general bacteriostatic effect on *Alternaria hydrophila*, and the main bacteriostatic substance is an extracellular product (Li et al., 2021). Consistent with previous studies (Chen et al., 2005; Li et al., 2021), the results of our study demonstrate that extracellular products have antifungal activity against *S. sclerotiorum*.

Bacillus is a beneficial bacterium, and a series of metabolites that can inhibit the activity of fungi and bacteria are produced during its growth. Most previous studies have focused on the direct antimicrobial characteristics of Bacillus or on the antifungal substances it produces (Li et al., 2016). However, few studies have explored the mechanisms underlying the fungal inhibitory effects of Bacillus spp. Therefore, we selected *B. amyloliquefaciens* and *S. sclerotiorum* for dual RNA sequencing analysis after inoculation with *S. sclerotiorum* alone*, B. amyloliquefaciens* alone, or with a mixture of the two. For *S. sclerotiorum*, the average FPKM of *S. sclerotiorum* transcripts in Gm-Ss and Gm-Ba-Ss was 117.82 and 50.79, respectively. Furthermore, for *S. sclerotiorum*, 507 upregulated and 4,950 downregulated genes were identified among all 8,975 genes in the paired comparison of Gm-Ba-Ss vs. Gm-Ss, and the number of downregulated genes was 9.76 times that of upregulated genes. These results suggest that *B. amyloliquefaciens* strongly inhibited gene expression in *S. sclerotiorum*.

**Figure 8:**
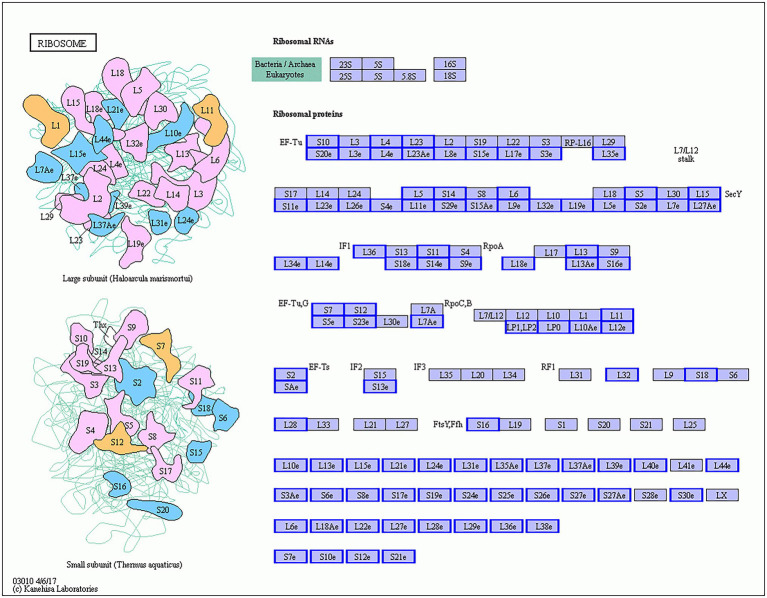
*S. sclerotiorum* differentially expressed genes (DEGs) in ribosome (ko03010) KEGG pathway. The genes with red/blue border in the figure were DEGs identified by dual RNA sequencing analysis, where red represents up-regulated genes and blue represents down-regulated genes.

Ribosomes are highly complex organelles mainly composed of ribosomal RNA and dozens of different ribosomal proteins. They mainly perform the function of protein synthesis and participate in the regulation of various biological processes. GO enrichment analysis of DEGs in Gm-Ba-Ss vs. Gm-Ss group revealed six significantly enriched and ribosome-related GO terms: ribosome (GO:0005840), structural constituent of ribosome (GO:0003735), ribosome biogenesis (GO:0042254), cytosolic ribosome (GO:0022626), ribosome assembly (GO:0042255), and preribosome (GO:0030684; Supplementary Table S8). Consistent with these results, all 5,457 DEGs in Gm-Ba-Ss vs. Gm-Ss group were significantly enriched in only one KEGG pathway, ribosome (ko03010; Table 1). Furthermore, all 95 DEGs in the ribosome (ko03010) KEGG pathway were downregulated, and they encoded proteins of the ribosome large and small subunits (Figure 8), including small subunit ribosomal protein genes *S10*, *L4*, *L23*, *S20e*, and *L17e* and small subunit ribosomal proteins *S7*, *S12*, *L11*, *S2*, and *L32*. Altogether, our results demonstrate that *B. amyloliquefaciens* strongly inhibited the expression of ribosomal protein-related genes in *S. sclerotiorum*, and interfered with ribosome biogenesis and ribosome assembly, thereby stopping growth and even causing death of *S. sclerotiorum.*

**Figure 9:**
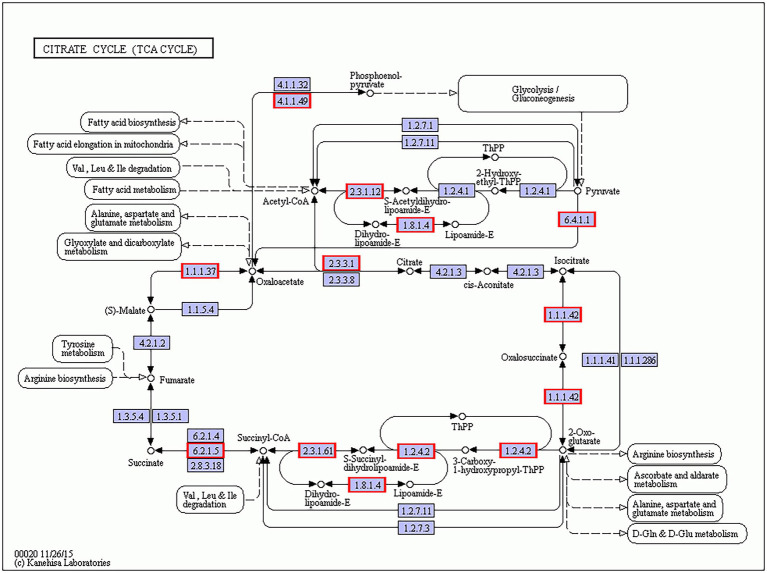
*B. amyloliquefaciens* differentially expressed genes (DEGs) in citrate cycle (ko00020) KEGG pathway. The genes with red/blue border in the figure were DEGs identified by dual RNA sequencing analysis, where red represents up-regulated genes and blue represents down-regulated genes.

As for *B. amyloliquefaciens*, the average FPKM of *B. amyloliquefaciens* transcripts in Gm-Ba and Gm-Ba-Ss treatments were 479.56 and 579.66, respectively. In the paired comparison Gm-Ba-Ss vs. Gm-Ba, 294 upregulated and 178 downregulated genes were identified among all 3,154 genes, and the number of upregulated genes was larger than that of downregulated genes. These results suggest that *S. sclerotiorum* induced overall gene expression in *B. amyloliquefaciens*. In this case, DEGs in Gm-Ba-Ss vs. Gm-Ba group were significantly enriched in 17 KEGG pathways, and 17 pathways were related to metabolism, including the citrate cycle (ko00020), carbon metabolism (ko01200), microbial metabolism in diverse environments (ko01120), butanoate metabolism (ko00650), and fatty acid degradation (ko00071). In the most significant case, i.e., the enriched TCA cycle pathway, all 11 DEGs were upregulated (Figure 9), including *pckA* (phosphoenolpyruvate carboxykinase ATP), *CS* (citrate synthase), and *MDH1* (malate dehydrogenase). pckA (KSO_RS05205) catalyzes the conversion of oxaloacetate to fructose-6-phosphate in the TCA cycle pathway (Medina et al., 1990). CS (KSO_RS05840) is involved in the first carbon oxidation in the TCA cycle pathway and catalyzes the conversion of oxaloacetate to 2-oxoglutarate (Goldenthal et al., 1998). MDH1 (KSO_RS05850) is involved in the second carbon oxidation in the TCA cycle pathway and catalyzes the conversion of 2-oxoglutarate to oxaloacetate (Goldenthal et al., 1998). The upregulation of these genes encoding important enzymes may indicate the activation of the TCA cycle pathway. The TCA cycle is a vital metabolic pathway for organisms and acts as a hub of aerobic metabolism. As an important metabolic pathway in bacteria, the TCA cycle generates precursors for the biosynthesis of many important substances and is also the most effective way to acquire energy for bacteria (Green and Emerich, 1997). Our study suggested that the pathway of the TCA cycle was enriched by gene upregulation, suggesting that the death or growth arrest of *S. sclerotiorum* promoted respiration and was beneficial for energy acquisition by *B. amyloliquefaciens.*

Based on the results of our study, we conclude that *B. amyloliquefaciens* can inhibit the expression of genes encoding the ribosomal subunits of *S. sclerotiorum*, thus resulting in the inhibition of fungal protein synthesis, which clearly seems to be the basis for the strong antagonistic effect of the bacterial strain on *S. sclerotiorum*. Protein biosynthesis is necessary for the survival of organisms. The inhibiting composition of *B. amyloliquefaciens* is an extracellular substance that can inhibit the expression of genes encoding the ribosomal subunits. It is speculated that the inhibiting effect of *B. amyloliquefaciens* should be broad-spectrum and have the potential to develop into an efficient fungicide. In addition to *S. sclerotiorum*, our research has already confirmed that *B. amyloliquefaciens* can also significantly antagonize growth of *Fusarium graminearum*, *Fusarium verticillioides*, *Exserohilum turcicum*, and *Fusarium oxysporum*. At present, the isolation and purification of the extracellular inhibiting component of *B. amyloliquefaciens* is still in progress. In the future, characteristics of the inhibiting component will be further studied, including heat resistance, drug resistance, and safety, which will be beneficial for promoting the application of the inhibiting component produced by *B. amyloliquefaciens*. Our study provides a scientific basis for the biological control of *S. sclerotiorum* and the rational utilization of *B. amyloliquefaciens*.

## Data Availability Statement

The data presented in the study are deposited in the SRA repository, accession number PRJNA824616. Our data have been released. You can get access our data using the link: https://www.ncbi.nlm.nih.gov/bioproject/PRJNA824616.

## Author Contributions

YC contributed to study conception and design, collection and/or assembly of data, and data analysis and interpretation. JL contributed to writing the manuscript. XG, HH, XZ, and RW prepared samples. All authors contributed to the article and approved the submitted version.

## Funding

This work was financially supported by the National Natural Science Foundation of China (No. 32172079) and the Department of Science and Technology of Jilin Province (No. 20200201113JC). The funding bodies had no role in the design of the study and collection, analysis, and interpretation of data and in writing the manuscript.

## Conflict of Interest

The authors declare that the research was conducted in the absence of any commercial or financial relationships that could be construed as a potential conflict of interest.

## Publisher’s Note

All claims expressed in this article are solely those of the authors and do not necessarily represent those of their affiliated organizations, or those of the publisher, the editors and the reviewers. Any product that may be evaluated in this article, or claim that may be made by its manufacturer, is not guaranteed or endorsed by the publisher.

## Footnotes

^1^ https://cutadapt.readthedocs.io/en/v2.7/

^2^ http://ccb.jhu.edu/software/hisat2/index.shtml

^3^ https://ccb.jhu.edu/software/stringtie/index.shtml

## Supplementary Material

Supplementary Table S2 Statistics information of raw reads and clean reads.

Supplementary Table S3 Alignment statistics of *Sclerotinia sclerotiorum*.

Supplementary Table S4 Alignment statistics of *Bacillus amyloliquefaciens*.

Supplementary Table S5 Transcript expression abundance of *Sclerotinia sclerotiorum*.

Supplementary Table S6 Transcript expression abundance of *Bacillus amyloliquefaciens*.

Supplementary Table S7 Sclerotinia sclerotiorum DEGs in comparison Gm-Ba-Ss vs Gm-Ss.

Supplementary Table S8 Bacillus amyloliquefaciens DEGs in comparison Gm-Ba-Ss vs Gm-Ba.

Supplementary Table S9 GO enrichment analysis of Sclerotinia sclerotiorum DEGs in paired comparison of Gm-Ba-Ss vs Gm-Ss.

Supplementary Table S10 GO enrichment analysis of Bacillus amyloliquefaciens DEGs in paired comparison of Gm-Ba-Ss vs Gm-Ba.
