# Supplementary material for: Dual RNA Sequencing Analysis of Bacillus amyloliquefaciens and Sclerotinia sclerotiorum During Infection of Soybean Seedlings by S. sclerotiorum Unveils Antagonistic Interactions
Source: Front Microbiol. 2022 Jun 23;13:924313. doi: 10.3389/fmicb.2022.924313 (PMC9260588; doi:10.3389/fmicb.2022.924313)
Supplement: Supplementary file 1 [file Data_Sheet_1.ZIP › ╕╜╝■/Table S1 Primers sequence information used in qRT-PCR.docx]

Table S1 Primers sequence information used in qRT-PCR

| *Bacillus amyloliquefaciens* | | | | |
| --- | --- | --- | --- | --- |
| Gene id | F | R | Length (bp) | |
| KSO_RS12540 | 5' cgcggaaagaaggggtgaa 3' | 5' gccgaacacagccgctaga 3' | 145 | |
| KSO_RS12550 | 5' gggggatgtcaagccgtttt 3' | 5' ccggcgagataggcatttaac 3' | 189 | |
| KSO_RS01495 | 5' gcgcaaactgggctatcc 3' | 5' gcccgtgaaaacctgtactg 3' | 115 | |
| KSO_RS03210 | 5' ggccgcgacgctgttatt 3' | 5' cccggtctcgtctgtttgaac 3' | 156 | |
| KSO_RS04805 | 5' gggaaatacggcggcacata 3' | 5' gccctgccatgttccgagtat 3' | 97 | |
| KSO_RS01805 | 5' gcccgatctcgtgcttttag 3' | 5' tccgagctcttttgattccttaa 3' | 151 | |
| KSO_RS01035 | 5' ggcgttttttatggagtctattt 3' | 5' tgccgtcaaaatccagaaaga 3' | 136 | |
| gyrA | 5' cggttcacagacggatttt 3' | 5' tgcgccattctgaccattgat 3' | 154 | |
| *Sclerotinia sclerotiorum* | | | |  |
| Gene id | F | R | Length  (bp) |  |
| SS1G_02985 | 5' ccgcgctcctccttcatg 3' | 5' gaggccgttggacttgagg 3' | 184 |  |
| SS1G_00067 | 5' tccccaagatacaacgtcaaga 3' | 5' gcctcctcgtggtccatgat 3' | 122 |  |
| SS1G_04031 | 5' ccggcatttgcacgagttc 3' | 5' ccgcaacccaaaccctcct 3' | 122 |  |
| SS1G_11420 | 5' cgcggacggggaaggac 3' | 5' gccggccatactcaacaatctc 3' | 121 |  |
| SS1G_13421 | 5' agcggtcgatgtggaagatt 3' | 5' ccggctgcgtcatacttaaat 3' | 95 |  |
| SS1G_00186 | 5' acccctcacctcgcagtctg 3' | 5' cggatggttcttcgggagtag 3' | 122 |  |
| SS1G_00103 | 5' tccgccagcacaccacatata 3' | 5' ggggtgatgcgcgaggttat 3' | 89 |  |
| ubiquitin | 5' tgcgcctccctccattaaa 3' | 5' cgcgggattgaaactcttg 3' | 106 |  |
